# Induction Apoptosis of Erinacine A in Human Colorectal Cancer Cells Involving the Expression of TNFR, Fas, and Fas Ligand *via* the JNK/p300/p50 Signaling Pathway With Histone Acetylation

**DOI:** 10.3389/fphar.2019.01174

**Published:** 2019-10-15

**Authors:** Ko-Chao Lee, Kam-Fai Lee, Shui-Yi Tung, Wen-Shih Huang, Li-Ya Lee, Wan-Ping Chen, Chin-Chu Chen, Chih-Chuan Teng, Chien-Heng Shen, Meng-Chiao Hsieh, Hsing-Chun Kuo

**Affiliations:** ^1^Division of Colorectal Surgery, Department of Surgery, Chang Gung Memorial Hospital, Kaohsiung Medical Center, Chang Gung University College of Medicine, Kaohsiung, Taiwan; ^2^Department of Information Management & College of Liberal Education, Shu-Te University, Kaohsiung, Taiwan; ^3^Department of Pathology, Chang Gung Memorial Hospital, Chiayi, Taiwan; ^4^Department of Hepato-Gastroenterology, Chang Gung Memorial Hospital, Chiayi, Taiwan; ^5^School of Medicine, Chang Gung University College of Medicine, Taoyuan, Taiwan; ^6^Division of Colon and Rectal Surgery, Department of Surgery, Chang Gung Memorial Hospital, Chiayi, Taiwan; ^7^Grape King Bio Ltd, Taoyuan, Taiwan; ^8^Department of Nursing, Chang Gung University of Science and Technology, Chiayi, Taiwan; ^9^Department of Surgery, Chang Gung Memorial Hospital, Chiayi, Taiwan; ^10^Graduate Institute of Clinical Medical Sciences, College of Medicine, Chang Gung University, Taoyuan, Taiwan; ^11^Research Center for Industry of Human Ecology, Chang Gung University of Science and Technology, Taoyuan, Taiwan; ^12^Chronic Diseases and Health Promotion Research Center, Chang Gung University of Science and Technology, Chiayi, Taiwan

**Keywords:** *H. erinaceus*, erinacine A, colorectal cancer cells, apoptosis, death receptors, JNK1/2, H3K9K14ac

## Abstract

Erinacine A, which is one of the major bioactive diterpenoid compounds extracted from cultured mycelia of *H. erinaceus*, displays great antitumorigenic activity. However, the molecular mechanisms underlying erinacine A inducing cancer cell apoptosis in colorectal cancer (CRC) remain unclear. This study found that treatment with erinacine A not only triggers the activation of extrinsic apoptosis pathways (TNFR, Fas, FasL, and caspases) but also suppresses the expression of antiapoptotic molecules Bcl-2 and Bcl-XL *via* a time-dependent manner in DLD-1 cells. Furthermore, phosphorylation of Jun N-terminus kinase (JNK1/2), NFκB p50, and p300 is involved in erinacine A–induced cancer cell apoptosis. Inhibition of these signaling pathways by kinase inhibitors blocks erinacine A–induced transcriptional activation implicates histone H3K9K14ac (Acetyl Lys9/Lys14) of the TNFR, Fas, and FasL as promoters. Moreover, histochemical and immunohistochemical analyses revealed that erinacine A treatment significantly induced the TNFR, Fas, and FasL levels in the *in vivo* xenograft mouse model. Together, these results demonstrated an increase in the cellular transcriptional levels of TNFR, Fas, and FasL by erinacine A induction to cell apoptosis *via* the activation of the JNK, p300, and NFκB p50 signaling modules, thereby providing a new mechanism for erinacine A treatment *in vitro* and *in vivo*.

## Introduction

Colorectal cancer (CRC), the most common cancer worldwide, is commonly categorized as a leading cause of cancer-related deaths due to its uncontrolled metastasis (Brenner et al., 2014). Very few CRC cases are confirmed by diagnosis at the early stage during the disease’s progression. Rapid tumor growth is a key feature in promoting the malignance of CRC along with poor outcome under medicinal therapies, such as surgery, chemotherapy, and radiotherapy (Jonker et al., 2007). Daily diet is an important risk factor for CRC, such as excessive red meat. On the other hand, treatment diet for colon cancer also has been considered as a vital way to prevent and fight CRC during and after medicinal therapies (Li-Weber, 2009). Thus, it is critical to find the novel diet compounds for the treatment of CRC.

**Figure 1:**
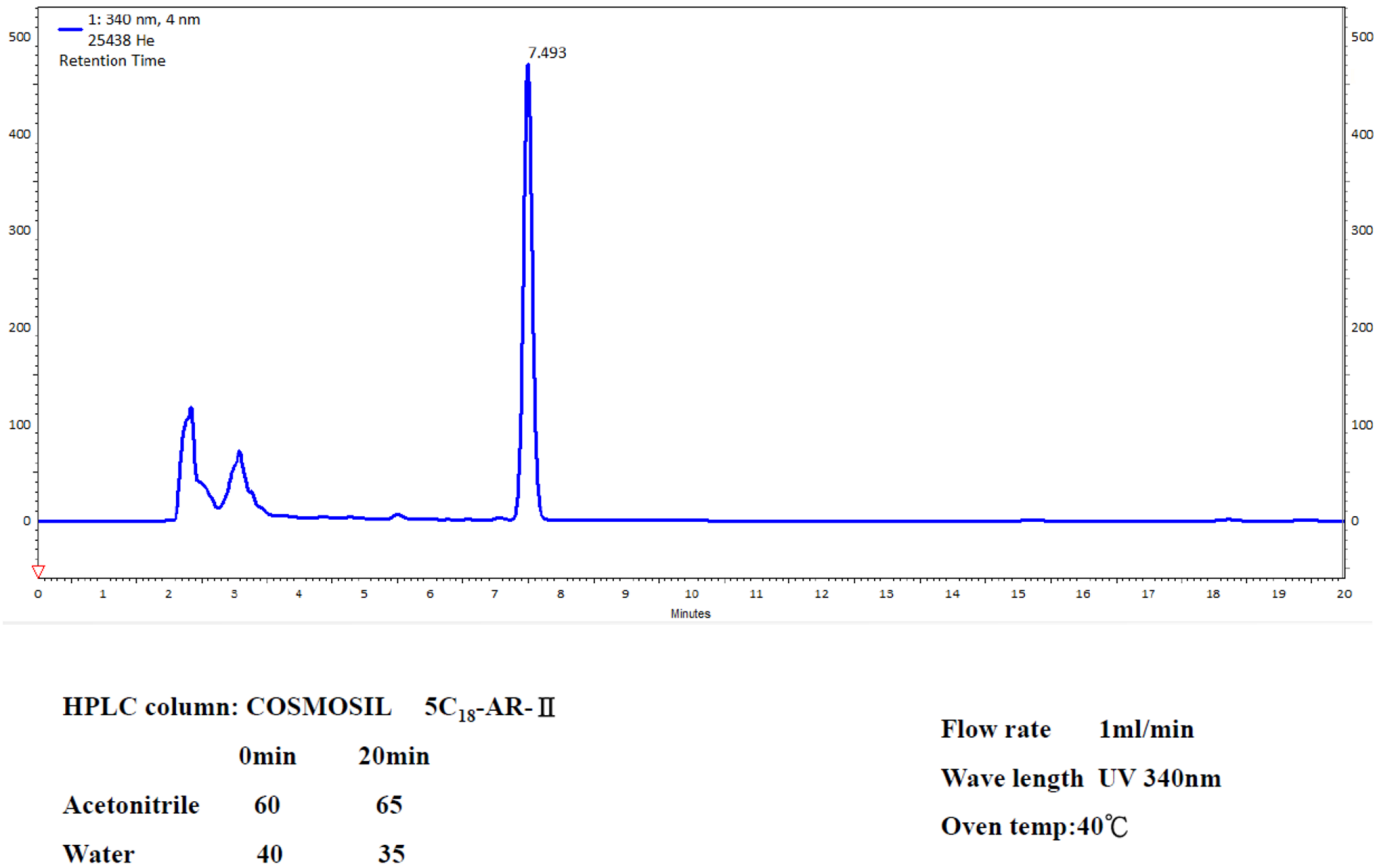
HPLC analysis of the ethanol *H. erinaceus* mycelium extract. For the conditions, see the Methods section. Retention time peak at 7.493 mins is erinacine A from 20-ton bioreactor (UV detection at 340 nm).

*Hericium erinaceus* (Lion’s mane or Yamabushitake), an edible mushroom with medicinal properties, is used as a culinary and medicinal product in Japan and China without harmful effects (Malinowska et al., 2009). As a candidate of traditional folk medicine, medicinal cuisine, and health-promoting compounds, the fruit bodies and mycelia of *H. erinaceus* contain a variety of structurally different components with valuable biological properties, such as the diterpenoid components (Ulziijargal and Mau, 2011). Erinacines A–I and hericenone C–H components are identified as a series of diterpenoid derivatives in the extracts of mycelium and the fruit bodies, respectively (Friedman, 2015). More recent studies have demonstrated that *H. erinaceus* possesses a number of therapeutic properties, including antioxidant activity (Han et al., 2013), hypolipidemic activity (Yang et al., 2003), hemagglutinating activity (Gong et al., 2004), antimicrobial activity (Yim et al., 2007), antiaging activity (Shimbo et al., 2005), and immune modulation and anticancer activities (Lee and Hong, 2010; Li et al., 2014). Erinacine A component (**Figure 1**), which previously has been collected and purified by ethanol extraction and HPLC analysis techniques from *H. erinaceus*, decreases malignance of several cancers, such as leukemia, hepatocarcinoma cancer cells, gastric cancer carcinomas, and CRC (Li et al., 2014; Kuo et al., 2016; Kuo et al., 2017). The evidence illustrates that cell cycle arrest and increased reactive oxygen species (ROS) production are involved in the erinacine A prevention against cancer cells proliferating and invasiveness through modulating PI3K/mTOR/p70S6K and ROCK1/LIMK2/Cofilin pathways (Kuo et al., 2017). In addition, our previous study exhibited that erinacine A induces CRC cells apoptosis and modulates the cancer-related actin depolymerization pathway, thereby inhibiting cancer invasion (Kuo et al., 2016). However, its molecular mechanism in inducing cell apoptosis of CRC remains unclear.

Impairment of cell apoptosis, which is an important physiological process of cell death, contributes to initiation, proliferation, growth, and aggressiveness of cancer (Brenner et al., 2014; Friedman, 2015). Cellular ROS generation is an intrinsic apoptotic stimulus that causes the release of cytochrome c from the mitochondria, resulting in the activation of caspase-9 and caspase-3 sequentially. Activated caspase-3 cleaves proteins, leading to apoptosis (Hanahan and Weinberg, 2000). On the other hand, the extrinsic pathway for apoptosis involves the binding of ligands Fas, FasL, and TNFR1 to their corresponding receptors, followed by the activation of caspase-8 and caspase-3 (Huang et al., 2017). Numerous studies have demonstrated that intracellular ROS function as the second messenger is sensitive to oxidative damage, in order to induce cell apoptosis under either intrinsic or extrinsic apoptotic stimulus (Li-Weber, 2013). Most recently, epigenetic modification such as histone acetylation is involved in selective dietary components-mediated death receptor-dependent apoptosis (Rajendran et al., 2011). In this study, we want to determine if erinacine A induces cell apoptosis of CRC at the epigenetic level and its mechanism. Our results showed that, in addition to activate JNK1/2, p300, and NFκB p50 signaling pathways, erinacine A increases the transcription activation of *TNFR*, *Fas*, and *FasL* genes through modulating histone H3 acetylation (Acetyl Lys9/Lys14) on their promoter areas, causing cell apoptosis of DLD-1 cells.

## Materials and Methods

### *Hericium Erinaceus* Extracts and Analysis of Erinacine A

*H. erinaceus* (BCRC 35669) was purchased from the Bioresources Collection and Research Center (BCRC) of the Food Industry Research and Development Institute (Hsinchu, Taiwan). The *H. erinaceus* was transferred from an agar slant into a potato dextrose agar plate and, then, maintained at 26°C for 15 days, as previously described (Li et al., 2014). After fresh mycelium extraction of *Hericium erinaceus* by ethanol, the fermentation process of the *Hericium erinaceus* mycelia was performed. Then, these mycelia were cultivated, harvested, lyophilized, ground to powder, and kept in a desiccator at room temperature. The mycelia extract was further concentrated and fractionated by a solvent partition between ethyl acetate and water. Following proximate composition analysis with silica gel column chromatography, HPLC analysis of erinacine A was executed according to the previous study with minor modifications (Kuo et al., 2016). By using the analytical COSMOSIL 5C18-AR-II column (250 × 4.6 mm; particle size 5 μm, Nacalai USA, Inc., Kyoto, Japan), the retention time of erinacine A was approximately ∼7.5 mins at a flow rate of 1.0 mL/min with a scanning UV wavelength at 340 nm. The yield rate of erinacine A in the *H. erinaceus* with ethanol extraction is ∼5 mg/kg, which was confirmed and quantified by HPLC (Kuo et al., 2016). The chemical compound of erinacine A (PubChem CID:9867477) is shown in **Figure 1**.

### Cell Culture

All culture materials were purchased from Gibco (Grand Island, NY, USA). Two human colon cancer cell line DLD-1 (CCL-221) and the human colorectal carcinoma cell line HCT-116 (CCL-247) were purchased from the American Type Culture Collection (ATCC). DLD-1 cells were cultured in RPMI 1640 medium composed of 10% fetal calf serum (FCS) (S0113; Biochrom KG, Berlin, Germany) and 1% antibiotics (100 units/mL of penicillin and 100 μg/ml of streptomycin); HCT-116 cells were cultured in DMEM supplemented with 10% heat-inactivated newborn calf serum. Passage number 1 of human normal human colonic epithelial cells (HCoEpiC) was purchased from ScienCell Research Laboratories (Carlsbad, CA) and cells were grown. Both cells were maintained at 37°C in a humidified 5% CO2 incubator (Lee et al., 2013).

### Cell Growth and Proliferation Assay

The MTT quantitative colorimetric assay is a method for cell viability determinations, as previously described (Kuo et al., 2017). The cells were incubated with MTT (0.5 mg/mL) for 4 h. After solubilization of the MTT-treated cells with isopropanol, the production of formazan was spectrophotometrically measured at 563 nm, which was directly proportional to the viable cells. The trypan blue (0.2%) exclusion assay (Huang et al., 2017) was performed to determine cell growth by manually counting the cells number with a Coulter counter at the indicated time points.

### Apoptosis Assay

The morphological characteristics of the cells stained with 4′,6-diamidino-2-phenylindole (DAPI) were observed under fluorescence microscopy. First, the cells were fixed with 4% paraformaldehyde for 30 mins at room temperature and, then, permeabilized in 0.2% Triton X-100 in phosphate-buffered saline three times for 15 mins. After PBS washing, these cells were incubated with 1 μg/ml of DAPI for 30 mins. Under 200× magnification using a fluorescent microscope with a 340/380 nm excitation filter, the percentage of the apoptotic nuclei in the field of the 200∼300 cells was observed and scored according to a previous report (Kuo et al., 2016).

Costaining with Annexin V–FITC and propidium iodide (Biosource International, USA) was used for measurement of cell apoptosis, as previously described (Huang et al., 2017). After staining, the cells were subjected to FACS analysis (Becton Dickinson), and the number of the apoptotic cells (V+/PI-) were quantified and analyzed by CellQuest and WinMDI software (Becton Dickenson). The data of fluorescent intensity are represented as a percentage of the untreated control group with three independent experiments.

### Protein Extraction and Immunoblot Analyses

The cells were lysed with a buffer, in which 1% NP-40, 0.5% sodium deoxycholate, 0.1% sodium dodecyl sulfate (SDS), and a protease inhibitor mixture (phenylmethylsulfonyl fluoride, aprotinin, and sodium orthovanadate) and the protein lysates were obtained, as previously described (Huang et al., 2016). Following SDS-polyacrylamide gel electrophoresis (PAGE) (12% running, 4% stacking) and transfer to the PVDF member, protein expression was detected by using specific antibodies, as indicated with Western-Light chemiluminescent detection system (Bio-Rad, Hercules, CA, USA).

### Animal Study

Animal care and the general protocols for animal experiments were approved by the Institutional Animal Care and Use Committee of Chang Gung Memorial Hospital, Chiayi, Animal Ethics Research Board (IACUC approval: 2012-017). Male BALB/c-nu nude mice, 4–6 weeks old (18-20 g), were purchased from the National Laboratory Animal Center in Taiwan and maintained under specific pathogen-free (SPF) conditions with sterilized food and water. The DLD-1 cells (106 cells/0.2 ml) were injected subcutaneously into the flanks of 4-week-old to 6-week-old female athymic BALB/c-nu mice. After tumor inoculation, the mice were randomly divided into four groups (n = 8 per group). The control group animals were treated daily with 0.1 mL DMSO (0.25%; i.p.); the test animals were treated with erinacine A at different concentrations of 1, 2, 5 mg/day; i.p. for 5 days. Tumor volumes were monitored and measured every four days using calipers. Calculation of tumor volumes was based on the following formula: length × width2 × π/6 ^14^. The body weights of the mice were measured every week to monitor drug toxicity. After 18 days of drug treatment, the mice were euthanized, and their tumors and organs, including the liver, lungs, and kidneys, were collected for further analysis.

### Histochemistry and Immunohistochemistry Analysis

Tumor tissue sections were fixed in 4% formaldehyde and, then, embedded in paraffin blocks. After staining with hematoxylin and eosin, these tissue slides were mounted for microscopic examination. Regarding immunohistochemical analysis, 5 μm thick sections of each subcutaneous tumor specimen were incubated with monoclonal anti-p21 and p70S6K antibodies (Santa Cruz, CA, USA) overnight at 4°C after blocking and, then, incubated with 1:100 diluted biotinylated horse antimouse IgG for 1 h. After the PBS wash, the tissue sections were reacted with 1:100 diluted avidin-biotin peroxidase mixture (Vectastain Universal Elite ABC Kit) for 30 mins. Following a thorough PBS wash, these slides were counterstained with hematoxylin, dehydrated, and mounted for microscopic examination. The digital images were captured using a digital camera (Canon A640), and the positive area and optical density (OD) of immunoreactive cells (brown) were analyzed in three randomly selected microscopic fields (400× magnification) for each slide. According to previous reports (Huang et al., 2016; Kuo et al., 2017), the IHC index was defined as having average integral optical density (AIOD; positive area × OD/total area).

### Chromatin Immunoprecipitation (ChIP) Analysis

DLD-1 cells were incubated with 1% formaldehyde at room temperature for DNA-protein crosslink, and then, 10 mins later, 125 mM glycine was added into the cells for 5 mins. The cells were scraped into an SDS lysis buffer (50 mM Tris-HCl [pH 8.1], 1% SDS, and 10 mM EDTA) and rotated with specific antibodies against with the histone H3K9K14ac and IgG overnight at 4°C in the presence of protease inhibitors (1 μg/ml leupeptin, aprotinin, and pepstatin A, 1 mM phenylmethylsulfonyl fluoride [PMSF]). After elusion with an elution buffer (50 mM Tris-Cl [pH 7.5], 1 mM EDTA, 1% SDS), the cross-links immunoprecipitated complexes were reversed at the temperature of 65°C incubation for at least 2 h. DNA fragments were purified by a ChIP DNA Clean & Concentrator Kit (Zymo), and then, quantitative polymerase chain reaction (PCR) analysis was performed to amplify the promoters region of the *TRAIL*, *DR5*, *Fas*, *FasL*, *TNFR*, and *TNFα* genes by using specific primers (**Table 1**) under the following conditions: 40 cycles of denaturation at 94°C, primer annealing at 60°C, and extension at 72°C. Disassociation curves were generated after each PCR to ensure that a single PCR product of the amplified appropriate length ran in electrophoresis. In addition, the mean CT ± SE was calculated from individual CT values from triplicate determinations per stage. The normalized mean CT was estimated as ∆CT by subtracting the mean CT of the input from that of the individual regions among the untreated control and drug treatment groups (Huang et al., 2012).

**Table 1:** The specific primers.

TRAIL
5′- TGCATGGATCCTGA GGGCAAGG -3′ 5′-TTGAACCTGCAACTGTCCCTCCC-3′
DR5
5′-GCCAGGGCGAAGGTTA-3′ 5′-GGGCATCGTCGGTGTAT-3′
FAS
5′-TTGGGTAACTTTGGGTGGTCC-3′ 5′-ATGTGGTTGGTTGTGAAGGGAG-3′
FasL
5′- GGGGGCAGTGTTCAATCTTA-3′ 5′- TGGAAAGAATCCCAAAGTGC-3′
TNFR
5′-GAT TGG TGG GTT GGG GGC ACA 5′-ATT AAA GCA GAG AGG AGG GGA GAG A
TNF-α
5′-CAA GCA TTA TGA GTC TCC GG 5′-AAG CTG TGT TGA GTC CTG AG

### Statistical Analysis

All data are expressed as the mean ± standard deviation and were compared between groups using the Student’s t-test or one-way analysis of variance (ANOVA) with Tukey’s multiple comparison test. The statistically significant difference between values was set at P < 0.05.14,35

## Results

### Effects of Erinacine A on the Viability of Human CRC DLD-1 Cells

**Figure 2:**
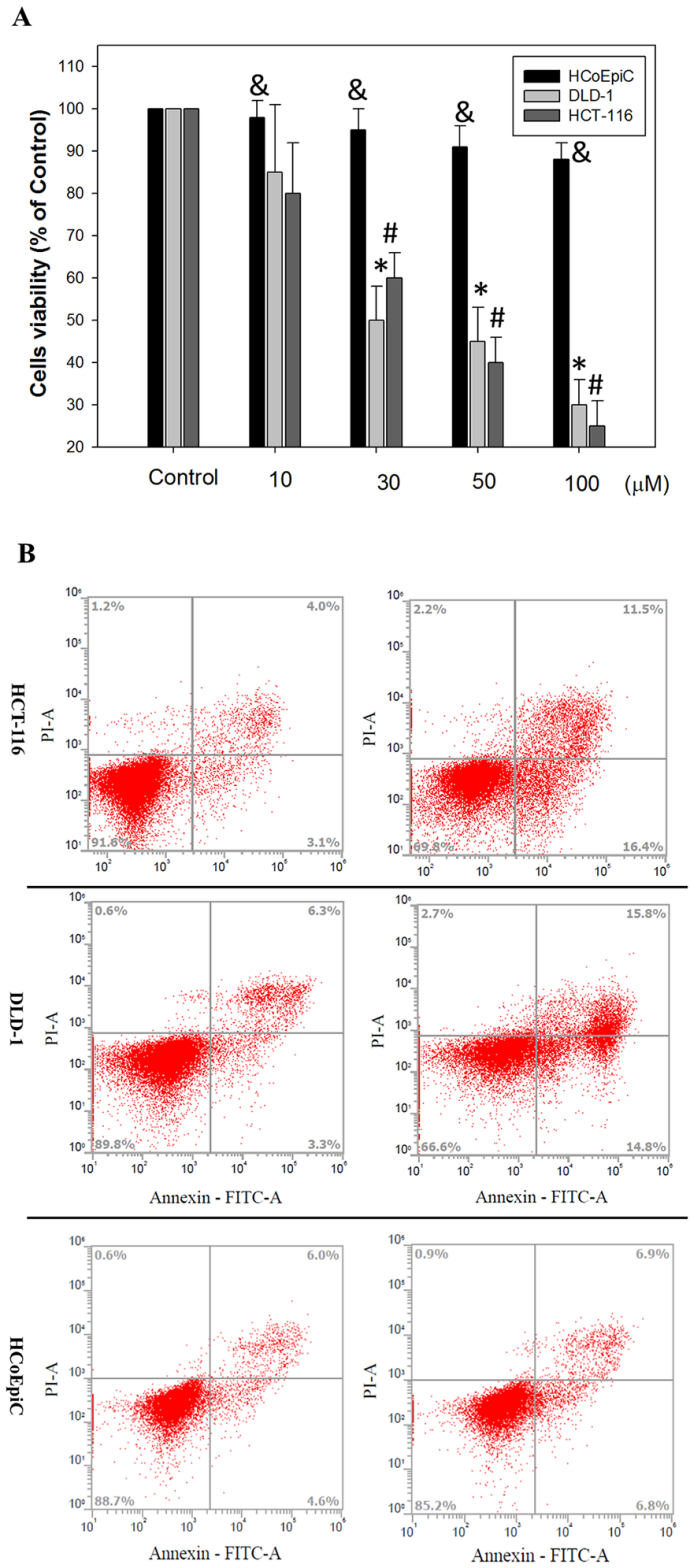
Effects of erinacine A on *in vitro* cell viability and the morphological characteristics of HCoEpiC, human colorectal cancer DLD-1 or HCT-116 cells, and its role in assessing cell death: **(A)** HCoEpiC and CRC cells were treated with either 0.1% DMSO (as the control) or erinacine A for 24 h, and the proportion of surviving cells was measured by the MTT assay; **(B)** HCoEpiC, HCT-116 and DLD-1 cells were treated with the vehicle or 30 μM erinacine A for 24 h, and cells were stained with FITC-conjugated Annexin-V and PI for flow cytometry analysis, as described in the Materials and Methods section. The data are presented as the mean of three repeats of one independent experiment. Other data in this figure are presented as the mean ± SD of three independent experiments. ^&^Indicates the means that are significantly different when compared to the control group (0.2% DMSO) of HCoEpiC with P < 0.05. *Indicates the means that are significantly different when compared to the control group (0.2% DMSO) of DLD-1 with P < 0.05. ^#^Indicates the means that are significantly different when compared to the control group (0.2% DMSO) of HCT-116 with P < 0.05.

The yield of purified erinacine A from the fresh mycelium of *H. erinaceus* by ethanol extraction and HPLC quantification was established in our previous study. HPLC analysis of erinacine A was executed according to the previous study with minor modifications. Separation was performed at 40°C using two different gradients for the mobile phase, which consisted of two solvents, acetonitrile (A) and water (B), with the following profile: 0–20 mins and the retention time of erinacine A was approximately ∼7.5 mins at a flow rate of 1.0 mL/min with a scanning UV wavelength at 340 nm (**Figure 1**). To determine whether the erinacine A had cytotoxic effects on human CRC cells, we treated HCoEpiC and human DLD-1 cells with erinacine A at different concentrations for 24 h and examined their cell viability by MTT assays. As shown in **Figure 2**, when treated with 30 µM, erinacine A was able to cause loss of the DLD-1 or HCT-116 cells by 53% and 60% reduction of human CRC viability with a dose-dependent manner but no cytotoxic effects of HCoEpiC cells. We further determined whether erinacine A reduced the cell viability of the HCT-116 and DLD-1 cells by inducing cell apoptosis. Our data showed that, after exposure to 30 µM erinacine A for 24 h, the untreated control group had 7 ± 2%, 9 ± 2% annexin V-positive cells, as basal control. After 24 h of erinacine A treatment, the annexin V-positive cells increased to 27 ± 2%, meaning 30 ± 2% in total cells, which is a characteristic feature for cell apoptosis (**Figure 2**).

### Activation of Extrinsic Cell Apoptosis Pathway by Erinacine A in DLD-1 Cells

**Figure 3:**
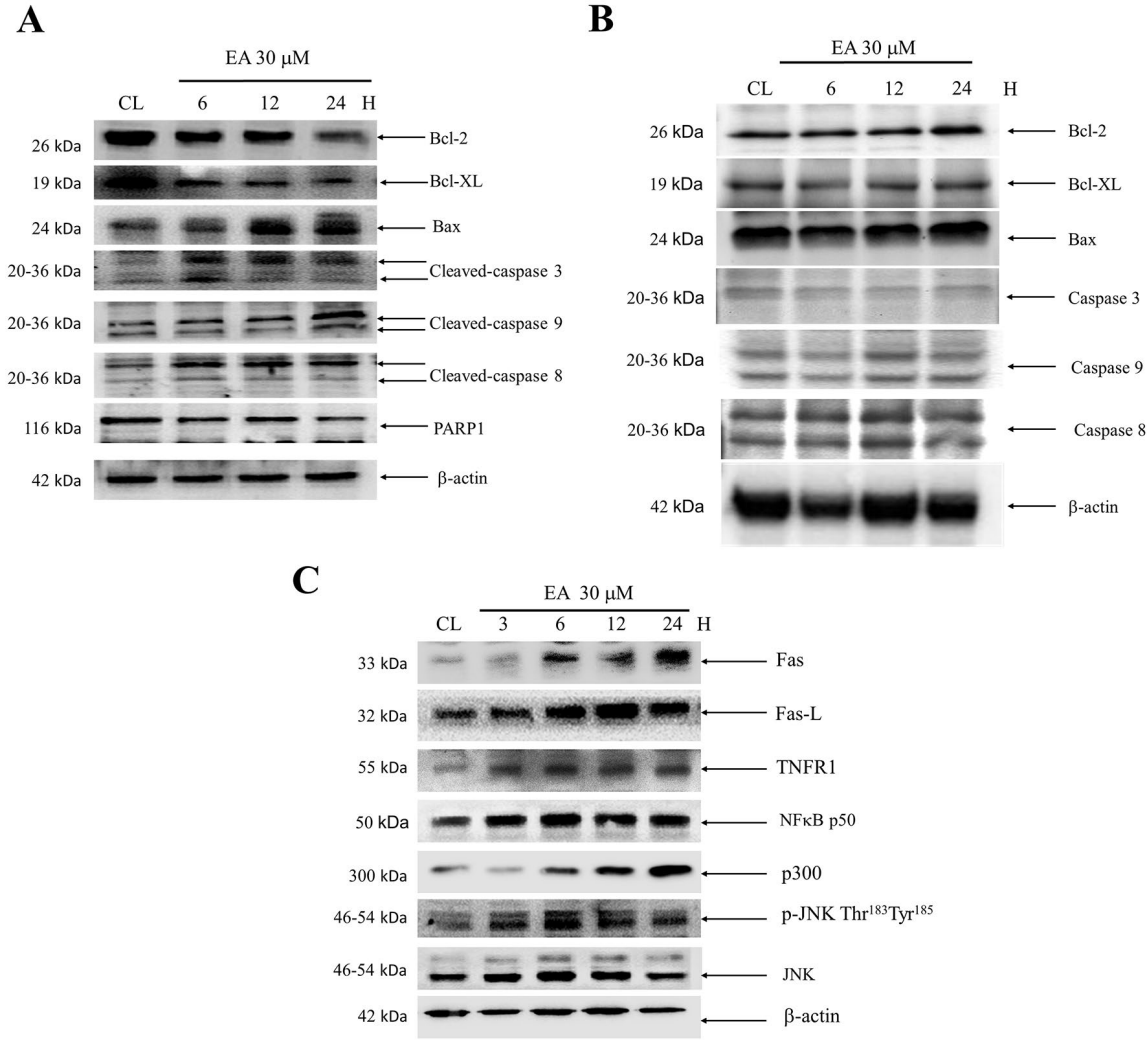
Effect of erinacine A on the Bcl-2 family of proteins, caspases, PARP1, Fas, FasL, TNFR1, and JNK/NFκB/p300 in HCoEpiC and HCT-116 or DLD-1 cells. Cells were treated with erinacine A for 3–24 h and separated by an SDS PAGE; subsequently, they were immunoblotted with antibodies against: **(A**, **B)** Bcl-2, Bcl-XL, Bax, FasL, cleavage caspase-3, cleavage caspase-9, cleavage caspase-8, and PARP1; and **(C)** Fas, FasL, TNFR, and p-JNK/NFkB/p300, or b-actin, which served as the internal control.

We determined whether erinacine A induced the DLD-1 cell apoptosis by regulating these cell death-related proteins. Western blot analysis revealed that erinacine A treatment increased the active form of caspase-3, -9, and -8 that has been classically considered as hallmarks of apoptotic cell death (**Figure 3A**). In addition, erinacine A also decreased the expression of the antiapoptotic proteins Bcl-2 and Bcl-XL but decreased the apoptotic protein Bax levels in the DLD-1 cells (**Figure 3A**). When assessment of erinacine A on *in vitro* cell viability of HCoEpiC cells and its role in assessing cell death and proteins expression, these findings suggest that erinacine A did not show significant cytotoxic effects in HCoEpiC cells (**Figure 3B**). Next, we determined if the extrinsic death receptor signaling pathway participated in erinacine A–induced cell apoptosis in DLD-1 cells. Our data showed that erinacine A treatment increased the protein level of Fas, FasL, and TNFR1 at 6, 12, and 24 h (**Figure 3C**). In addition, phosphorylated JNK at Thr183 and Tyr185, NFκB, and p300 protein were upregulated in DLD-1 cells treated with erinacine A (**Figure 3C**).

### Activation of the Intrinsic Cell Apoptosis Pathway by Erinacine A in DLD-1 Cells

**Figure 4:**
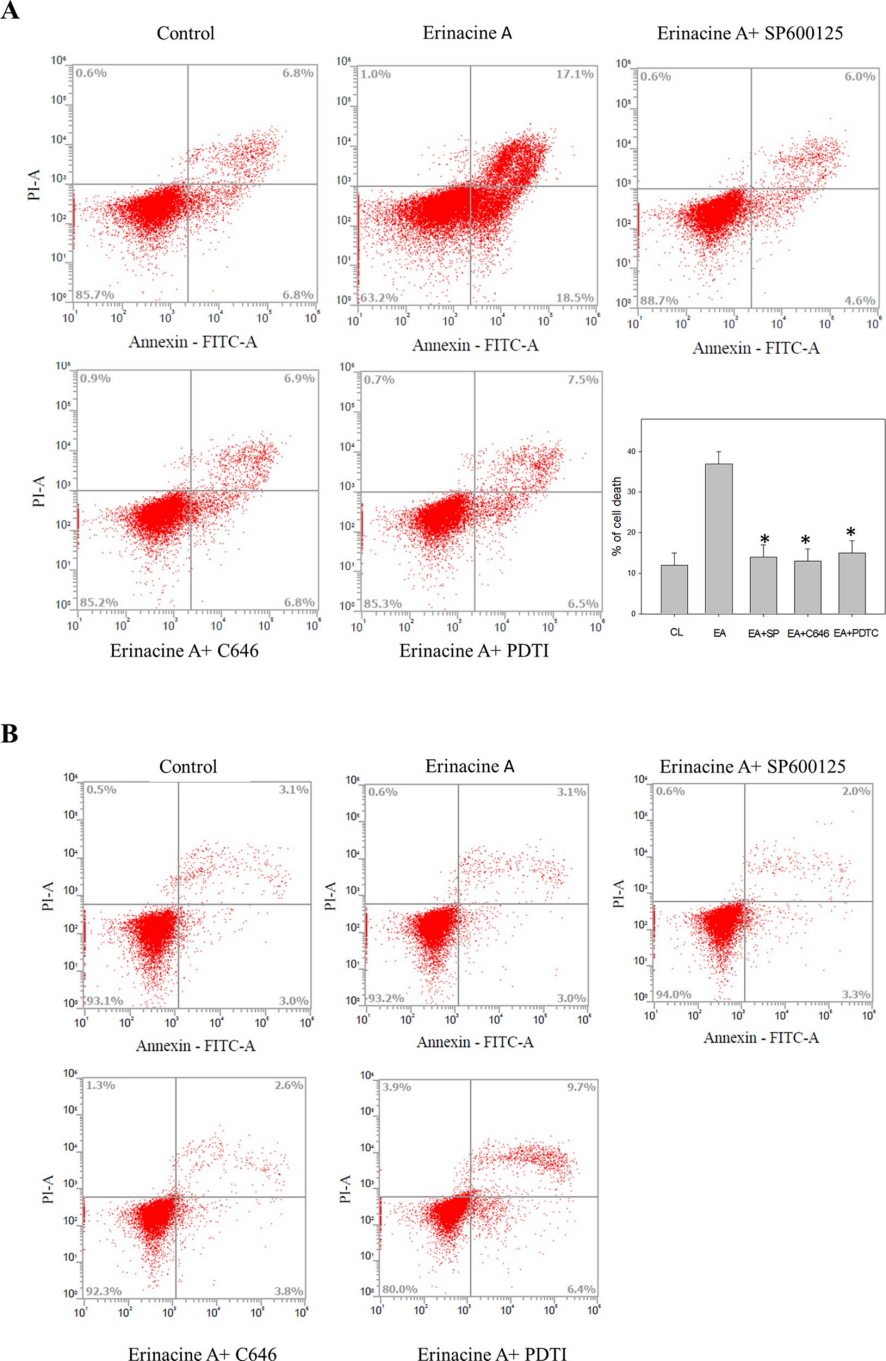
Effects of the kinase inhibitors blocking the erinacines A induction associated with DLD-1 cells death. After the indicated treatment for 24 h, the DLD-1 **(A)** and HCoEpiC cells **(B)** were stained with FITC-conjugated Annexin-V and PI for flow cytometry analysis, as described in the Materials and Methods section. The percentages presented in each frame depict the apoptotic cells. *P <0.01, as compared with the control group (0.2% DMSO).

**Figure 5:**
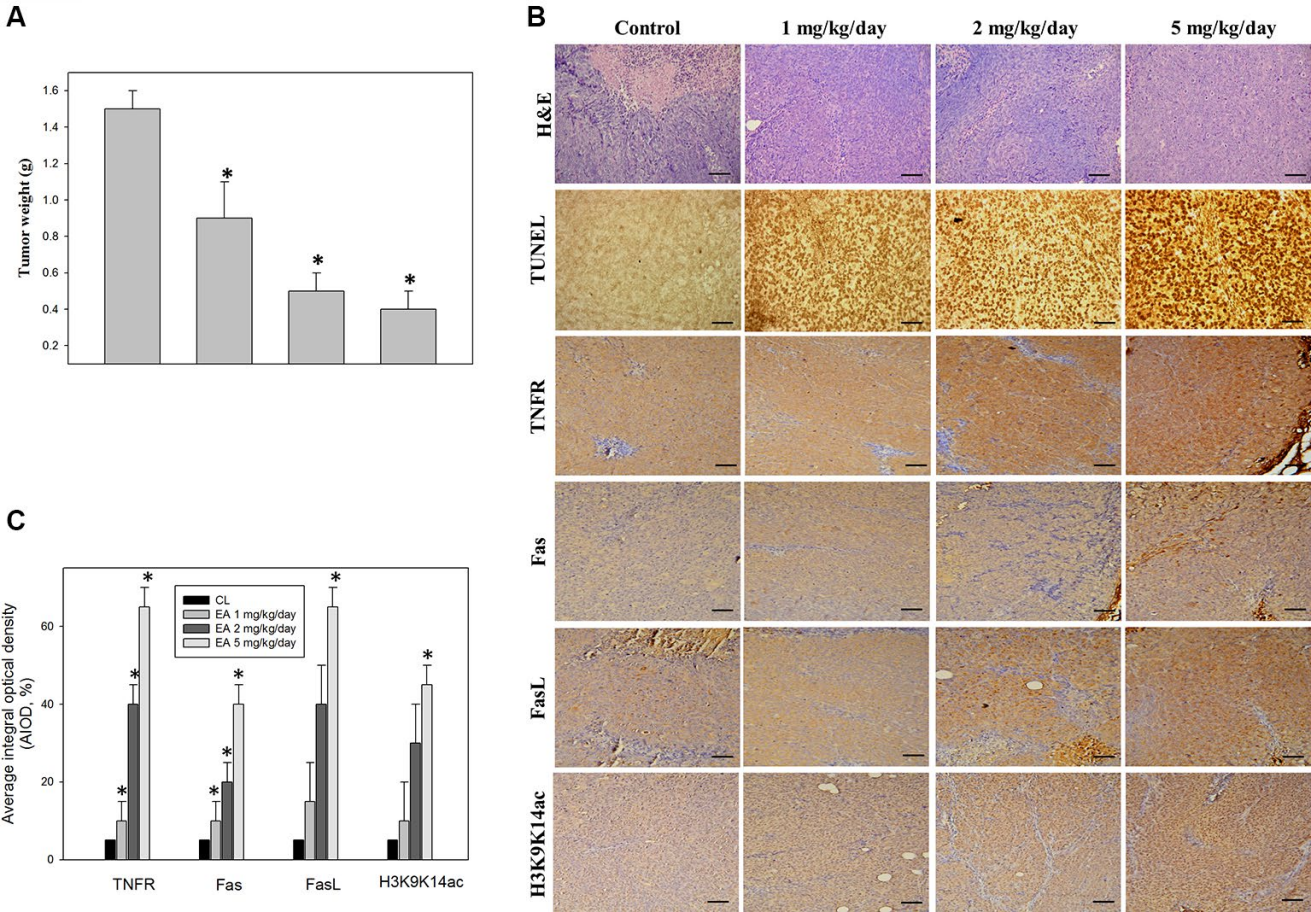
Immunohistochemical analysis of tumor inhibition of the CRC xenograft by erinacine A. Nude mice were implanted subcutaneously with DLD-1 cells into their flanks on day 0 and, then, treated with or without (as a control) erinacine A, as described in the Materials and Methods section. **(A)** The results are presented as isolated tumors and tumor weights. **(B)** Effect of erinacine A on the growth of the DLD-1 xenograft was evaluated by immunohistochemical analysis of the tumors was conducted, and multiple tumor fields were evaluated per group. Representative images for all groups from both experiments are presented. H&E staining revealed similar s.c. tumor morphology among all groups of tumors. Fas, FasL, and TNFR staining show the expressed tumor cells treated with erinacine A. TUNEL staining revealed significantly greater apoptosis in response to erinacine A in tumors. **(C)** Quantitative immunohistochemical proteins Fas, FasL, TNFR, and histone H3K9K14ac were evaluated by average integrated optical density (AIOD). The positive stained area was evaluated from three randomly selected observation fields of each brain section. Data are expressed as mean ± SD (n = 6/group). *P < 0.05, as compared with the control group at magnification X400.

Annexin-V staining was used to verify that treatment with erinacine A can induce DLD-1 cells apoptosis. Our data showed that the untreated control group had 12 ± 2% annexin V-positive cells, as basal control. After 24 h of erinacine A treatment, the annexin V-positive cells increased to 36 ± 2 in total cells (**Figure 4**). Consistent with **Figure 3**, our data showed that erinacine A treatment elicited DLD-1 cells apoptosis. Several signaling pathways involved in the induction of cell apoptosis and activation of caspase can be triggered by either the intrinsic (mitochondria-mediated) or extrinsic (receptor-mediated) stimuli, including Bcl-2, Bcl-XL, Bax, NFκB, p300, c-Jun N-terminal kinases (JNK), and p38 mitogen-activated protein kinase (p38MAPK) (Khanal et al., 2011; Teng et al., 2012). To investigate the roles of the JNK1/2 and the NFκB p50/p300 signaling pathways in erinacine A–induced DLD-1 cells apoptosis, we exposed DLD-1 cells to erinacine A and then cotreated them with the specific JNK inhibitor SP600125, p300 inhibitor C646, or the NFκB p50 inhibitor (PDTC). The effects of those inhibitors in blocking erinacine A–induced cell death were determined, and then, erinacine A did not show significant cytotoxic effects in HCoEpiC cells, which were demonstrated no effect of apoptosis signaling pathway by the kinase inhibitors (**Figure 4**). Furthermore, we examined if erinacine A also can induce DLD-1 cells apoptosis *in vivo* xenograft mouse model. Erinacine A at the concentrations of 1, 2, and 5 mg/kg/day was intraperitoneally injected into the nude mice xenografted with DLD-1 cells. **Figure 5** shows that the tumor volume of DLD-1 xenograft of the erinacine A-treated (1, 2, 5 mg/kg/day) mice was inhibited to 60, 32, and 26%, as compared with the control group after cell implantation. At the end of the experiment, DLD-1 xenograft tumor of each mouse was removed and weighed. It demonstrated that erinacine A significantly decreased the solid tumor mass as compared to the control group (**Figure 5**). In addition, no signs of toxicity were observed (body weight and microscopic examination of individual organs; data not shown) in all nude mice. H&E and TUNEL staining revealed that intraperitoneal injections of erinacine A reduced the tumors along with an increase in the number of cell deaths in these tumors compared with the untreated control group (CL) (**Figure 5**). Consistent with the *in vitro* results, immunohistochemistry staining analysis revealed that the number of TNFR1, Fas, and FasL positive cells was significantly increased by erinacine A at the concentrations of 2 and 5 mg/kg (**Figure 5**, middle and bottom panels; *P < 0.05).

### JNK MAPK/p300/NFκB p50 Pathways Involved in the Regulation of Erinacine A–Induced Transcriptional Activation of Histone H3K9K14ac (Acetyl Lys9/Lys14) of TNFR, Fas, and Fas-L Promoters in DLD-1 Cells

**Figure 6:**
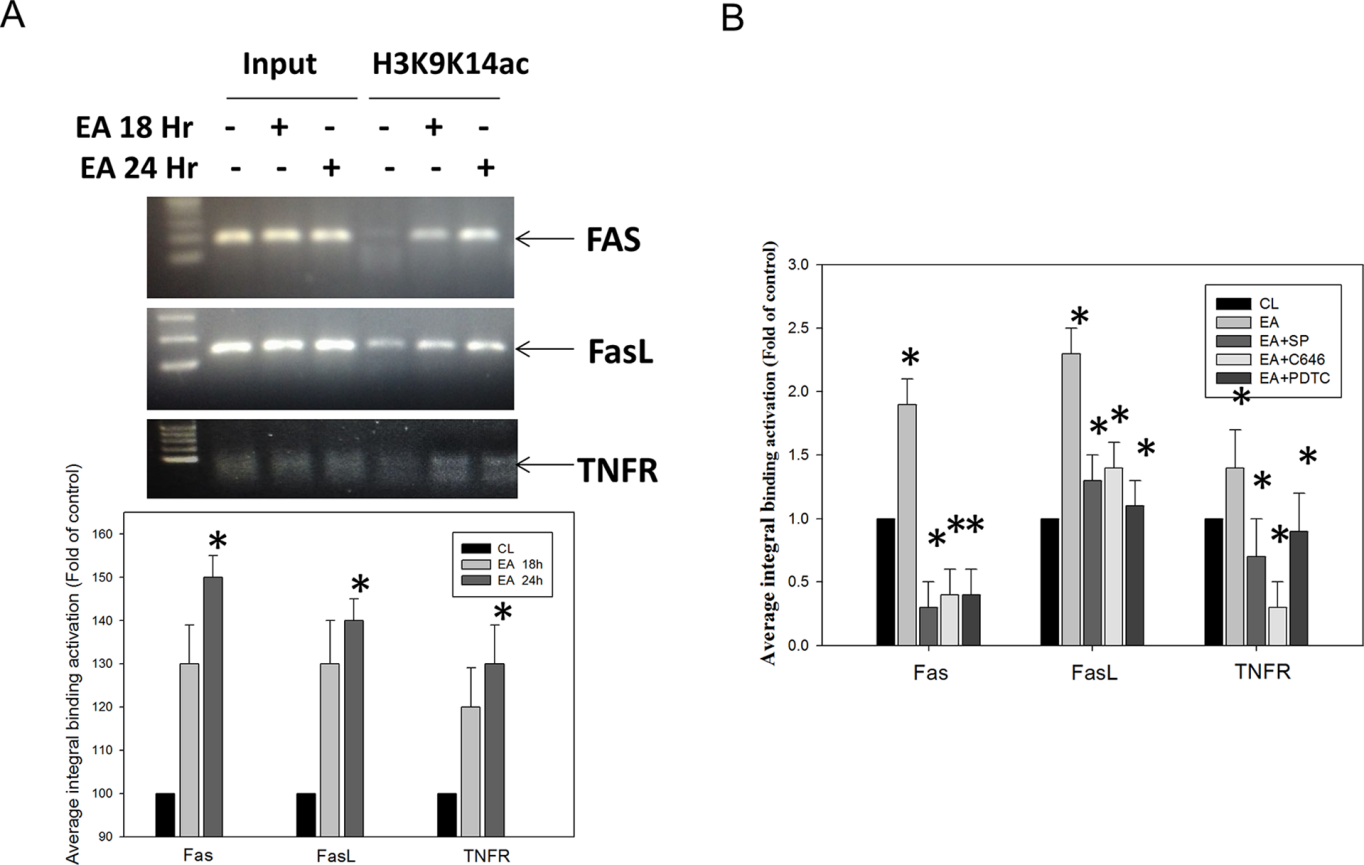
Effect of the kinase inhibitors in blocking the binding activities of the Fas, FasL, and TNFR promoter regions, as induced by erinacine A stimulation. **(A)** Next, the chromatin immunoprecipitation (ChIP) assays were performed using antibodies against histone H3K9K14ac (Acetyl Lys9/Lys14), in order to pull down associated DNA. The precipitated DNA was amplified by PCR using primer sets specific to the target sites of Fas, FasL, and TNFR promoters. DNA pulled down by the anti-IgG antibody served to identify the background amplification. Input DNA was amplified as a loading control. The recovered DNA is amplified by quantitative real-time PCR (qRT-PCR), which used specific primers on the sequence of interest to detect the bound DNA in electrophoresis. The CT values, as generated in triplicate technical repeats, are similar, and the quantitative data are presented as the mean of three repeats of one independent experiment. **(B)** DLD-1 cells were incubated with or without various concentrations of the specific JNK1/2 inhibitor SP600125, NFκB inhibitor PDTC, or the p300-Binding Protein inhibitor C646 for 24 h. The data are presented as the mean ± SD of three independent experiments. *P < 0.05, as compared to the control group.

Epigenetic modification of the genes involved in cell growth, proliferation, and apoptosis has been implicated in pathogenesis of CRC (Bannister and Kouzarides, 2011). For instance, histone H3 acetylation on K9 and K14 (histone H3K9K14ac) signifies the well-established markers of active gene transcription. To determine if histone H3K9K14ac participated in the erinacine A-upregulated gene expression, we first checked the level of histone H3K9K14ac in the tumor areas in the *in vivo* xenograft mouse model with erinacine A injection. Our data showed that erinacine A injection increased the level of histone H3K9K14ac measured by immunohistochemistry staining (**Figure 5**). Furthermore, we determined if erinacine A can alter the status of histone acetylation on the promoters of *Fas*, *FasL*, and *TNFR* genes by ChIP with anti-H3K9K14ac (Acetyl Lys9/Lys14) antibodies. The PCR primers specific for the promoter regions of Fas, FasL, and TNFR were used. Our data showed that treatment of DLD-1 cells with erinacine A increased the level of histone H3 acetylation on the promoters of Fas, FasL, and TNFR at 24 h (**Figure 6**). Some reposts demonstrate that activation of JNK, p300, and NFκB signaling is a critical event for the upregulation of gene expression by histone acetylation (Behrens et al., 2001; Wang et al., 2015). Thus, we studied if JNK, p300, and NFκB were involved in the modification of histone H3K9K14ac by erinacine A. Specific chemical inhibitors SP600125, C646, and PDTC were used to inhibit the JNK, p300, and NFκB activation, respectively. In **Figure 6**, our results indicate that inhibition of JNK, p300, and NFκB activation by these inhibitors all decreased the erinacine A–induced promoter acetylation of Fas, FasL, and TNFR on histone 3 at K9 and K14 residuals (H3K9K14ac (Acetyl Lys9/Lys14)). Taken together, these results suggest that erinacine A treatment upregulated the death receptor molecules, such as Fas, FasL, and TNFR through JNK MAPK/p300/NFκB pathway-mediated histone H3K9K14ac modification, in order to induce CRC apoptosis.

## Discussion

CRC is one of the most commonly occurring malignant tumors in the digestive tract. The main or first treatment for CRC that has not spread to distant sites is usually surgery; chemotherapy may also be used after surgery (Haggar and Boushey, 2009). Usage of natural or synthetic substances is considered as additional chemoprevention following cancer treatment to prevent cancer formation or cancer progression. Some medicinal herbs or foods are potential sources of chemopreventive compounds for antitumor activities that target the apoptosis pathways in cancer cells (Li-Weber, 2009; Fullgrabe et al., 2011). Here, we found the novel functions of *H. erinaceus* on inducting CRC apoptosis, in part, through the epigenetic modification of the death receptor-dependent signaling pathways. *H. erinaceus* has long been used for its beneficial health properties. Our *in vitro* data demonstrated that erinacine A and the treatment concentration of 30 µM resulted in a significant cytotoxic effect against human colorectal DLD-1 or HCT-116 cancer cells (**Figure 2**). Furthermore, erinacine A treatment for 24 h resulted in an induction of DLD-1 cell apoptosis. Erinacine A induces sustained activation of the JNK1/2 and p50/p300 pathway, and the apoptotic pathway is required for erinacine A induction of DLD-1 cells apoptosis (**Figure 4**). We also found that erinacine A induced apoptosis on other HCT-116 cells (unpublished data). Previous studies have shown that a number of dried fruit body *Hericium erinaceus* extractions could reduce the expression of MMP-2 and MMP-9 in human colon cancer and invasion through modulations of the phosphorylation of ERK, JNK, and p38 MAPK (Kim et al., 2011; Kim et al., 2013; Lee et al., 2014). Our study demonstrated that *H. erinaceus* mycelium erinacine A treatment at the concentration of 30 µM for 24 h resulted in an induction of DLD-1 cell apoptosis, which caused the activation of caspase-3, caspase-9, and caspase-8 in the time-dependent induction of apoptosis and a decrease in the Bcl-2 and Bcl-XL levels (**Figure 3**). In addition, there was an increase in the cellular levels of the phospho-JNK MAPK/p300/NFκB p50 pathways, TNFR, Fas, and FasL in the erinacine A–induced apoptosis (**Figure 3**). Moreover, the present *in vivo* study demonstrated that intraperitoneal injections of erinacine A (1–5 mg/kg/day) treatment significantly increased the expression of TNFR, Fas, and FasL, as well as histone H3K9K14ac (Acetyl Lys9/Lys14). This was examined using immunohistochemistry in the DLD-1 CRC xenograft of nude mice (**Figure 5**).

Growing evidence indicates that these pathways could strongly contribute to preventing cancer growth and that, when induced, they could sustain MAPK activation, leading to cell death by dietary phytochemicals. They are widely present in food and nutraceuticals (Li et al., 2014). Phytochemicals have shown the cellular changes that cause modulation of the MAPK pathways by inducing apoptosis (Rajendran et al., 2011; Teng et al., 2012). These may be a promising target for anticancer effects (Li-Weber, 2009; Li-Weber, 2013). Our previous results showed that erinacine A could be used to investigate *in vitro* and *in vivo* antitumor activity through cell cycle arrest in human DLD-1 cancer cells involved in the generation of the ROS activates p70S6K/NFκB pathways, leading to p21 expression. These activation effects result from the phosphorylation of the PI3K/mTOR/p70S6K and ROCK1/LIMK2/Cofilin pathways, as well as the execution of apoptosis and antiinvasiveness by erinacine A (Kuo et al., 2016; Kuo et al., 2017). Additionally, other JNK/p300/NFκB p50 pathways were directly involved in inducing apoptosis by *H. erinaceus* mycelium erinacine A in this study.

Previous data suggest that natural phytochemicals from certain plants that affect the epigenome can also trigger sustained DNA damage and apoptosis induction^31^. Examples are cited from *in vitro* and *in vivo* studies of polyphenols, isothiocyanates, epigallocatechin-3-gallate (EGCG), curcumin, resveratrol sulfur, selenium compounds, indoles, sesquiterpene lactones, and anacardic acid (Kouzarides, 2007; Rajendran et al., 2011). It is interesting that they can induce the growth arrest of neoplastically transformed cells and trigger apoptosis *via* signaling pathways in cancer cells exposed to dietary phytochemicals, such as histone deacetylase inhibitors (HDAC) (Bolden et al., 2006). Inhibition of HDAC activity may occur in human colon cancer cells, with an increase in histone H3 acetylation in extensive histone modification statuses, such as induction of *TNFR*, *Fas*, *FasL*, and *p21* genes, *via* histone acetyltransferase (HAT) p300 (Bolden et al., 2006; Kouzarides, 2007; Rajendran et al., 2011). Similarly, for the first time, our current *in vitro* study demonstrated that erinacine A treatment significantly upregulated the expression of p300 and H3K9K14ac (Acetyl Lys9/Lys14) in DLD-1 cells, as well as transcriptional activation of histone H3K9K14ac (Acetyl Lys9/Lys14) of the TNFR, Fas, and FasL promoters (**Figure 6**). Thus, erinacine A, as an individual natural phytochemical, may be seen as a novel chemotherapeutic agent worth continued investigation in the treatment of CRC. Additional studies are still needed to elucidate the erinacine A effects on the HAT and HDAC between different molecular cellular signaling pathways and epigenetic machinery, as well as to determine *in vivo* CRC cells’ xenograft.

## Conclusion

**Figure 7:**
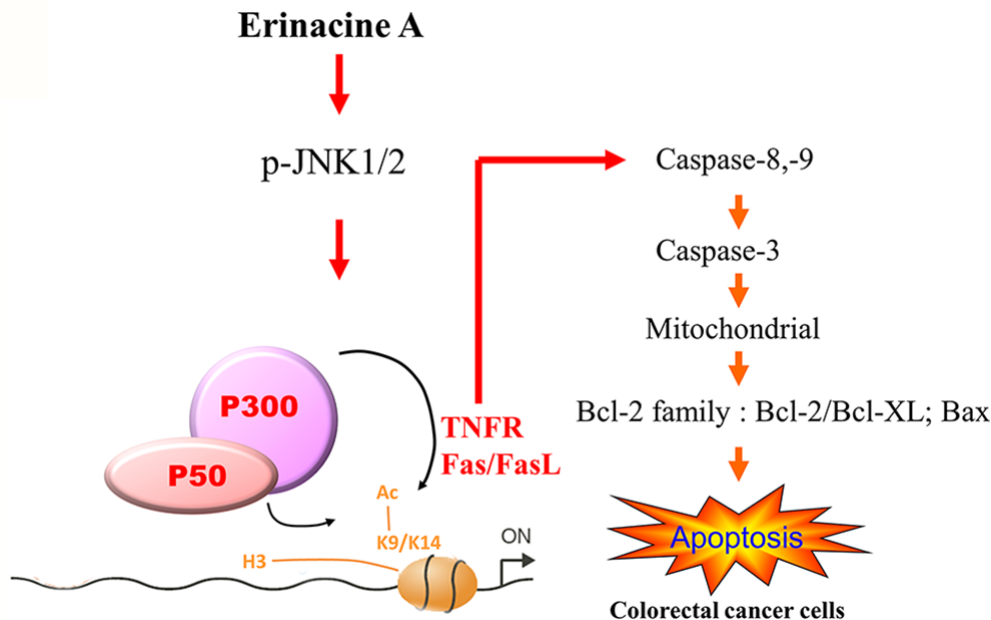
Schematic presentation of the signaling pathways involved in erinacine A–induced cell apoptosis in human DLD-1 cancer cells, and the effect of erinacine A on the activation of the JNK/p300/p50 NFκB pathways, which leads to TNFR, Fas, and FasL expression with the implications of histone H3K9K14ac (Acetyl Lys9/Lys14) in human DLD-1. Erinacine A triggered the apoptosis pathway through the inhibition of Bcl-2 and Bcl-XL and the Bax increase and activation of caspase-8, -3, and -9.

In conclusion, this study suggests the roles of *H. erinaceus* mycelium components, erinacine A–induced apoptosis, and the histone modification (H3K9K14ac) of the TNFR, Fas, and FasL promoters by the JNK/p300/NFκB p50 signaling pathways in human DLD-1 cancer cells. These results led us to theorize that erinacine A may play a role in an apoptotic cascade in DLD-1 cells *via* TNFR, Fas, and FasL, and Bax, the inhibition of Bcl-2 and Bcl-XL expression and cytochrome c release, and caspase-8, -9, and -3 activation (**Figure 7**). This study is especially interesting with regard to the antitumor effect of erinacine A as it relates to the development of novel dietary phytochemicals for the *H. erinaceus* mycelium and epigenetic mechanism in the treatment of malignant CRC.

## Data Availability Statement

The raw data supporting the conclusions of this manuscript will be made available by the authors, without undue reservation, to any qualified researcher.

## Ethics Statement

Animal care and the general protocols for animal use were approved by the Institutional Animal Care and Use Committee of Chang Gung Memorial Hospital, Chiayi, Animal Ethics Research Board (2013-014). All procedures and the reporting thereof comply with the Institutional Animal Care and Use Committee (IACUC) guidelines.

## Author Contributions

K-CL: provision of study material, collection and assembly of data and histopathological evaluation, and manuscript writing; W-SH: conception, collection, and assembly of data; C-HS: provision of study material or animals; K-FL: provision of study material or animals pathology; S-YT: provision of study material or animals; C-CT: provision of study material, collection, and assembly of data; K-CL: administrative support, collection, and assembly of data (flow cytometry); L-YL, C-CC, and W-PC: provision of study material or animals; M-CH and H-CK: conception and design, financial support, administrative support, manuscript writing, and final approval of manuscript. All authors read and approved the final manuscript.

## Funding

The funding for this study was provided in part by research grants from the Chang Gung Memorial Hospital, Chiayi, Taiwan. This study was supported by grants BMRPD42, CLRPG8G0591, CMRPG8G0531, CMRPG8G0532, CMRPF6G0011, CMRPF6G0012, and CMRPF6G0013, CMRPF6I0021, from Chang Gung Memorial Hospital, Chiayi, Taiwan, and Chang Gung University of Science and Technology, Chia-Yi Campus, Taiwan, and by the Ministry of Science and Technology, Taiwan (MOST 108-2622-B-255 -001 -CC3 and MOST 107-2320-B-255 -001 -MY3).

## Conflict of Interest

L-YL, W-PC, and C-CC were employed by Grape King Bio Ltd.

The remaining authors declare that the research was conducted in the absence of any commercial or financial relationships that could be construed as a potential conflict of interest.

## Abbreviations

Acetyl Lys9/Lys14, H3K9K14ac; ChIP, Chromatin immunoprecipitation assays; HAT, histone acetyltransferase; HDAC, histone deacetylase inhibitors; JNK1/2, Jun N-terminus kinase; p38 MAPK, p38 mitogenactivated protein kinase; ROS, reactive oxygen species.
